# Analyzing the antibacterial effects of food ingredients: model experiments with allicin and garlic extracts on biofilm formation and viability of *Staphylococcus epidermidis*

**DOI:** 10.1002/fsn3.199

**Published:** 2015-02-14

**Authors:** Xueqing Wu, Regiane R Santos, Johanna Fink-Gremmels

**Affiliations:** Division Veterinary Pharmacology, Pharmacotherapy and Toxicology, Faculty of Veterinary Medicine, Institute for Risk Assessment Sciences, Utrecht UniversityUtrecht, The Netherlands

**Keywords:** Allicin, ATCC 12228, ATCC 35984, bacterial biofilm, *Staphylococcus epidermidis*

## Abstract

To demonstrate different effects of garlic extracts and their main antibiotic substance allicin, as a template for investigations on the antibacterial activity of food ingredients. *Staphylococcus epidermidis* ATCC 12228 and the isogenic biofilm-forming strain ATCC 35984 were used to compare the activity of allicin against planktonic bacteria and bacterial biofilms. The minimal inhibitory concentration (MIC) and the minimum biofilm inhibitory concentration (MBIC) for pure allicin were identical and reached at a concentration of 12.5 *μ*g/mL. MBICs for standardized garlic extracts were significantly lower, with 1.56 and 0.78 *μ*g/mL allicin for garlic water and ethanol extract, respectively. Biofilm density was impaired significantly at a concentration of 0.78 *μ*g/mL allicin. Viability staining followed by confocal laser scanning microscopy showed, however, a 100% bactericidal effect on biofilm-embedded bacteria at a concentration of 3.13 *μ*g/mL allicin. qRT-PCR analysis provided no convincing evidence for specific effects of allicin on biofilm-associated genes. Extracts of fresh garlic are more potent inhibitors of *Staphylococcus epidermidis* biofilms than pure allicin**,** but allicin exerts a unique bactericidal effect on biofilm-embedded bacteria. The current experimental protocol has proven to be a valid approach to characterize the antimicrobial activity of traditional food ingredients.

## Introduction

Garlic (*Allium sativum*) is recognized as a medicinal herb for more than 5000 years and truly deserves to be described as functional food. Its technical and potential medical use are described in more than 4400 scientific articles and cover medical applications as an antibiotic described already by Louis Pasteur, and more recently the use of stabilized garlic extracts in the prevention of cardiovascular diseases, diabetes, hyperlipidaemia, chronic inflammation, and as antiaging and anticancer remedy (Amagase 2006; Borlinghaus et al. 2014). In food production, garlic has been used not only as flavoring agent, but particularly as preservative for meat and meat products (Aguirrezábal et al. 2000; Yin and Cheng 2003; De Moura Oliveira et al. 2005; Mariutti et al. 2008; Cao et al. 2013). Due to its antioxidant capacity garlic prevents rancidity and exerts antimicrobial activity prolonging the shelf life of meat products and increasing food safety due to its effect against zoonotic pathogens such as Staphylococci, *Salmonella* spp., and *Escherichia coli* (Ankri and Mirelman 1999). The antimicrobial activity of garlic extract, particularly the water-soluble ingredients such as allicin, have gained recently increasing attention as they are active against emerging pathogens such as methicillin-resistant *Staphylococcus aureus* (MRSA) (Cutler and Wilson 2004) and plaque-forming organisms in the oral cavity, which may be associated with garlic consumption (Bachrach et al. 2011). The dental plaques contain bacteria in a biofilm stage. A biofilm can be described as sessile community of bacteria, surrounded by a shelf-produced extracellular matrix that adheres the cells to biological and artificial surfaces. Within a biofilm, bacteria are temporary in a dormant stage, which conveys resistance to antibiotics as in dormancy the molecular targets for antibiotics such as cell wall synthesis, DNA, and protein-synthesis are largely suppressed (Burmølle et al. 2010). The ability of bacteria, including almost all common pathogens, particularly those residing on the skin or mucosal surfaces, to form biofilms has revived the interest in herbs, as plants are known to express substances with antibiofilm/antifouling properties.

Here, we describe a series of experiments conducted with allicin, the major antimicrobial compound in garlic, and allicin-containing aqueous and ethanol extracts resembling the ingestion of garlic as food additive and herb in a normal diet. As a model organism to study the antibacterial and antibiofilm effects, we chose *Staphylococcus epidermidis*, from which two well-characterized isogenic type strains are available denoted ATCC 12228, a strain growing in planktonic cultures, and ATCC 35984 (also described as RP62A) that rapidly forms biofilms under in vitro culture conditions (Fey and Olson 2010). Both type strains were used to test the effects of allicin and allicin-containing garlic extracts on bacterial viability, biofilm formation, and the survival of bacteria within a biofilm. The ultimate aim of this study was to provide a template for the assessment of commonly used herbs and related plant products in human (and animal) diets.

## Materials and Methods

### Chemicals

Allicin was purchased from LKT (LKT Laboratories Inc., St. Paul, MN) at a concentration of 10.2 mg/mL in methanol/water (40/60) with 0.1% formic acid and stored at −20°C. This original solution was diluted 200 times in tryptone soya broth (TSB) + 0.25% glucose (TSB^+^) to obtain the working solution of 50 *μ*g/mL allicin. Therefore, the final concentrations of methanol and formic acid were 0.1% and 0.00025%, respectively. At these concentrations, the solvents did not affect biofilm formation or bacterial viability (Wu et al. 2014). All other chemicals used in the present study were purchased from Sigma-Aldrich (St. Louis, MO) at the highest available purity.

### Preparation of garlic extracts

Extracts of fresh garlic obtained in a normal grocery were prepared with water (garlic water extract; gWE) or with pure ethanol (garlic ethanol extracts; gEE). The gWE mixture consisted 15 g garlic bulbs, which were fragmented and milled in 0.2 mol/L phosphate buffer (PBS) (pH 6.5) for 10 min, mixed for 1 h after adding 60 mL deionized water at room temperature and centrifuged for 10 min at 3076 *g* at room temperature (Sigma 3-16K). The precipitate was discarded and the supernatant was kept at 4°C before use. gEE was also obtained from commercially available garlic bulbs as described before (Yang et al. 2009) with minor modifications. In brief, 15 g of the garlic bulbs were fragmented and milled in an aliquot 0.2 mol/L PBS (PH6.5) for 10 min at room temperature, and then 60 mL of 95% ethanol (fresh garlic: ethanol/1:4 w/w) was added to this mixture. This suspension was kept in a thermostatic water bath for 1.5 h at 30°C and was then filtered through Watman paper filters and evaporated to absolute dryness to exclude any effects of ethanol residues. In the obtained extracts, allicin was quantified by HPLC according to the method of Ilic et al. (2012). Intra- and interday precision of the method was within the acceptable limits with relative standard deviation (RSD) <5%. The limit of quantification was 0.1 *μ*g/mL. Working solutions of these extracts in the microbiological assays were standardized for the allicin content.

### Bacterial strains and culture conditions

Two strains of *S. epidermidis* were purchased from the American Type Culture Collection (ATCC) (Mercatorstr. 51, Wesel, Germany). The ATCC 35984 is known for its ability to form biofilms, while the strain ATCC 12228 is unable to form biofilm under experimental conditions (Fey and Olson 2010) (Fig. S1A and B). Both strains were maintained on tryptone soya agar (TSA) (Oxoid CM 129) (Scheepsbouwerweg 1B, Landsmeer, Netherlands) slants at 4°C. One colony of bacteria was cultured in 10 mL TSB^+^ (pH 7.0) under aerobic conditions at 37°C for different times according to different experiments.

### Minimum inhibitory concentration of allicin on *S. epidermidis* in planktonic cultures

To identify the concentration range for the following biofilm experiments, initially the minimum inhibitory concentration (MIC) of allicin was determined using the nonbiofilm-forming ATCC 12228 strain, following the Clinical and Laboratory Standards Institute (CLSI) Standard Broth Micro-dilution Method with minor modifications. Briefly, serial twofold dilutions of 50 *μ*g/mL allicin in TSB^+^ down to a final concentration of 0.195 *μ*g/mL allicin were prepared in a U-bottom 96-well plate (100 *μ*L per well). To each well, 100 *μ*L of 10^6^ CFU/mL of the bacterial suspension was added, resulting in a final volume of 200 *μ*L and final concentrations of allicin of 0.098, 0.195, 0.39, 0.78, 1.56, 3.13, 6.25, 12.5, or 25 *μ*g/mL, respectively. Wells with sterile TSB^+^ alone served as blanks. Plates were incubated at 37°C for 24 h. Thereafter, OD values were measured at 655 nm wavelength after transferring 100 *μ*L of the incubated suspension to a new sterile flat-bottom 96-well plate. MIC was defined as the lowest allicin concentration resulting in OD value similar to blank (TSB^+^). To allow a comparison with data from the bacterial viability in the biofilm, next to this standard broth dilution assay, a viability staining was conducted with SYTO® 9 green (green staining of total bacteria) and propidium iodide (red staining of bacteria with membrane damage; nonviable) (Molecular Probes Europe, Leiden, the Netherlands).

### Bacterial inoculum and time-dependent biofilm formation

To establish the experimental conditions for the biofilm assays, a series of experiments were conducted to determine the optimal inoculum size as well as the incubations times required for the formation of a stable biofilm. Results of these assays are presented as supplementary data (Figs. S1A, S2) and revealed that an inoculum size of 10^6^ CFU/mL is requested to achieve a dense biofilm of ATCC 35984 and that maximum biofilm density could be measured after 24 h.

### Determination of the minimum biofilm inhibitory concentration (MBIC)

Biofilm formation was assessed by the standard safranin colorimetric assay as described earlier (Melchior et al. 2006; Wu et al. 2014) using the strain ATCC 35984. In brief, 100 *μ*L of the bacterial suspension (10^6^ CFU/mL) was transferred into each well of a U-bottom 96-well microtiter polystyrene plates (Costar, Corning, NY). To this suspension gWE and gEE or pure allicin was added dissolved in broth to reach allicin concentrations of 0.195, 0.39, 0.78, 1.56, 3.13, 6.25, 12.5, 25, or 50 *μ*g/mL resulting in final tested concentration in the samples of 0.098–25 *μ*g/mL allicin. Wells with sterile TSB^+^ alone served as blanks. The plates were incubated on a microplate shaker (Heidolph titramax 100) (Matsonford Road 100, PA, US) at 37°C for 24 h. At the end of culture period, the supernatant from all wells was discarded and the biofilms adhered to the bottom of the wells were incubated with 200 *μ*L 0.1 mol/L HCl for 1 h at room temperature. Thereafter, HCl was replaced by safranin (0.1% in water) and the plates incubated for 45 min at room temperature. Nonbound safranin was removed by rinsing the wells three times with deionized water, and thereafter plates were incubated with 125 *μ*L 0.2 mol/L NaOH per well at 57°C for 1 h. At the end of incubation, 100 *μ*L from the stained dissolved biofilm in each well was pipetted to a new flat-bottom 96-well microtiter polystyrene plate and its intensity was measured at a wavelength of 540 nm in a microplate reader. The minimum biofilm inhibitory concentration (MBIC) was defined as the lowest concentration that inhibited at least 90% biofilm formation. Each test was performed in quadruplicate with three independent repetitions.

### Bacterial viability within biofilms determined by confocal laser scanning microscopy

Biofilms of ATCC 35984 formed on coverslips, which were inserted into tubes containing either control medium or medium supplemented with gWE and gEE or allicin at the standard range of test concentrations (0.098–1.56 *μ*g/mL allicin) were evaluated. After culture, coverslips carrying bacterial biofilms were incubated for 15 min in the dark at 37°C with 1 nmol/L SYTO® green fluorescent nucleic acid dye and 6 nmol/L propidium iodide. After being labeled, biofilms were washed three times in PBS and finally examined using confocal laser scanning microscopy (CLSM) (Leica TCS SPE-II, Mannheim, Germany). Bacteria in the biofilm were classified as nonviable if stained positively by propidium iodide (red). Image generation was achieved using the 488 and 543 nm wavelengths for SYTO® green and propidium iodide, respectively. To estimate the percentages of nonviable bacteria, the program Image J 1.4.7 (NIH, Bethesda, MD, US) was used to count the propidium-stained cells (given as cell area).

### Quantitative RT-PCR

Along with the biofilm formation assay, samples were taken at the end of incubation and submitted to RNA isolation. For this, 1 mL of bacterial suspension was centrifuged for 10 min at 4°C 15,000 *g*. The supernatant was removed and 1 mL Trizol reagent was added, mixed and then the suspension was transferred to a Lysing Matrix E tubes (MP Biomedicals Germany GmbH, Eschwege, Germany) and homogenized in the FP120 Cell Disrupter (Thermo Savant, Qbiogene, Inc., Cedex, France) for 45 sec at speed of 6.5 m/sec two times. Subsequently, the samples were centrifuged for 5 min at 15,000 *g* 4°C, and the supernatants were separately transferred to 1.5 mL Eppendorff tubes and subjected to the phenol-chloroform RNA extraction protocol. The concentration and purity of total RNA were spectrophotometrically assessed using a NanoDrop 1000™ (Thermo Scientific, Waltham, MA), and 1 *μ*g of extracted total RNA from each sample was reverse transcribed with the iScript™ cDNA Synthesis kit (Bio-Rad, Hercules, CA) according to the instructions of the manufacturer. The obtained cDNA was diluted to a final concentration of 30 ng/mL. Primers (Table S1) complementary to *S. epidermidis* were designed according to literature, and were commercially produced (Eurogentec, Maastricht, the Netherlands). The primers were selected based on specificity and efficiency by qPCR analysis of a dilution series of pooled cDNA at a temperature gradient (55–65°C) for primer-annealing and subsequent melting curve analysis. The reaction mixture for the qPCR contained 10 *μ*L diluted cDNA, 12.5 *μ*L iQSYBR Green Supermix (Bio Rad Laboratories Inc.), forward and reverse primers (final concentration of 0.4 pmol/*μ*L for each primer), and sterile water according to the manufacturer's instructions. qPCR was performed using the MyiQ single-color real-time PCR detection system (Bio-Rad) and MyiQ System Software Version 1.0.410 (Bio Rad Laboratories Inc.). The mRNA quantity was calculated relative to the expression of two reference genes, hsp60 and tpi (Table S1).

### Statistical analysis

For the biofilm formation assay and bacterial viability test, data were evaluated using one-way analysis of variance (ANOVA) by Prism 6.04 (La Jolla, CA, US). For qRT-PCR test, down- and upregulation were considered significant when the relative expression was decreased or increased ≥4 folds. All experiments were repeated at least three times.

## Results

### Minimum inhibitory concentration (MIC) of allicin on *S. epidermidis* ATCC 12228 in planktonic cultures

**Figure 1:**
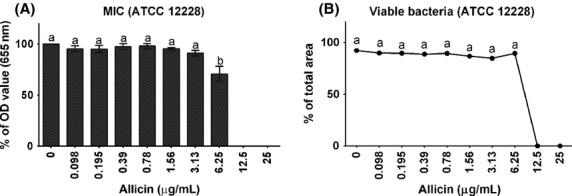
Minimum inhibitory concentration (mean ± SEM) of allicin determined in *Staphylococcus epidermidis* ATCC 12228 growing in planktonic cultures. (A) Results obtained with the standard CLSI broth dilution protocol; (B) Results (mean ± SEM) obtained with a live/dead staining and quantification of dead and live bacteria with confocal microscopy. Different lower-case letters (a, b) indicate significant differences (*P *< 0.001) between allicin concentrations.

Using the standard broth microdilution protocol, we could determine a MIC value of 12.5 *μ*g/mL for *S. epidermidis* ATCC 12228, despite the observed a slight reduction in OD values already at a concentration of 6.25 *μ*g/mL allicin (Fig.1A). This value was confirmed using a live/dead staining with SYTO® green and propidium iodide and a quantitative evaluation of confocal microscopy images (Fig.1B).

### Time- and concentration-dependent effects of allicin on biofilm formation

**Figure 2:**
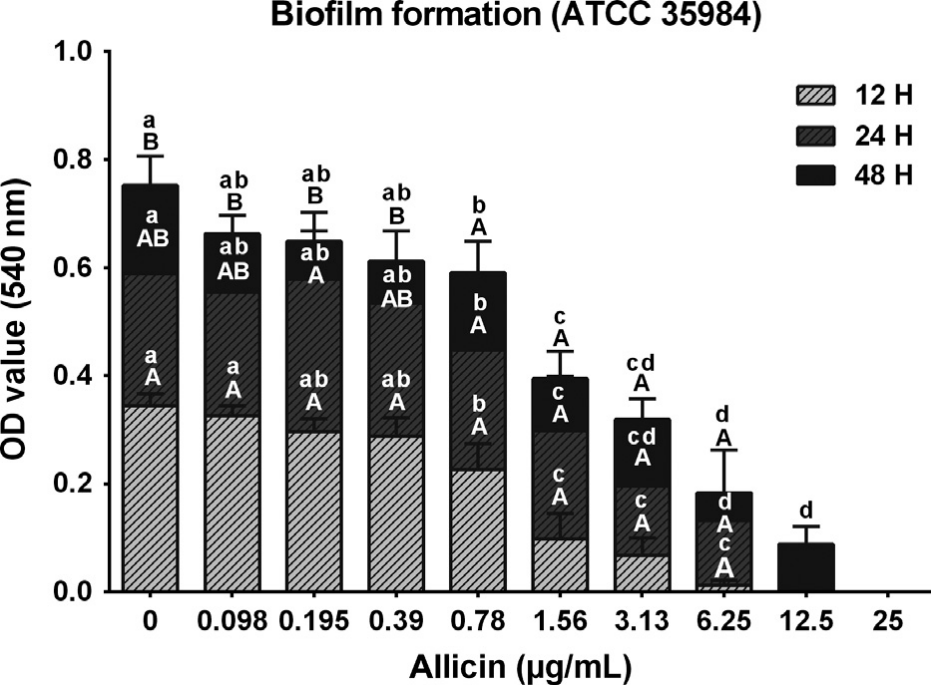
*Staphylococcus epidermidis* (ATCC 35984) biofilm formation within 48 h and after exposure to different concentrations (0.098–25 *μ*g/mL) of allicin. Different upper-case letters (A, B) indicate significant (*P <* 0.001) differences between time points within the same allicin concentration. Different lower-case letters (a–d) indicate significant (*P <* 0.0001) differences between allicin-treated groups within the same time point.

Using the safranin staining protocol to assess biofilm formation by *S. epidermidis* (ATCC 35984) after 12, 24, and 48 h, respectively, it could be shown that allicin inhibits biofilm formation in a concentration-dependent manner (Fig.2). In the tested concentration range varying from 0.78 to 25 *μ*g/mL, significant differences in comparison to the controls were observed already during the initial 12 h of culture. Biofilm biomass was increasing during the following 12 h significantly, whereas between 24 and 48 h only a slight further increase was observed. Based on these results, 24 h biofilms were used for the forthcoming experiments on biofilm sensitivity and architecture.

### Minimum biofilm inhibitory concentration and bacterial viability in the biofilm

**Figure 3:**
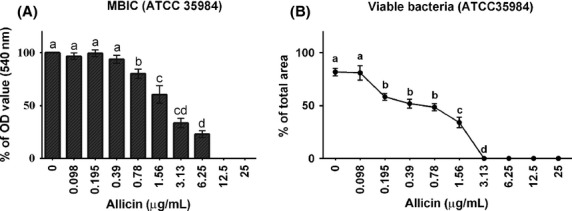
Minimum biofilm inhibitory concentration (MBIC) and viability of *Staphylococcus epidermidis* (ATCC 35984) within a biofilm exposed to different concentrations of allicin. (A) Results of the safranin staining method (% OD value at 540 nm wavelength, mean ± SEM). (B) Results of live/dead staining with SYTO® green and propidium iodide and quantitative evaluation with CLSM (mean ± SEM). Different letters (a–d) indicate significant (*P <* 0.0001) differences.

*Staphylococcus epidermidis* (ATCC 35984) was cultured in the presence of different concentrations of allicin for 24 h to determine the MBIC. The MBIC is defined as the concentration at which biofilm density is reduced by >90% when compared with OD values of controls (here at an OD value <0.06). Using this definition, it could be shown that the MBIC of allicin was 12.5 *μ*g/mL, but biofilm formation was inhibited significantly already after exposure to 0.78 *μ*g/mL allicin (Fig.3A). The percentage of viable bacteria in the biofilm showed a rapid and significant decrease (*P <* 0.0001) to 60% of controls already after exposure to 0.195 *μ*g/mL allicin, and exposure to 3.13 *μ*g/mL allicin resulted in a complete loss of viability (100%) of bacteria within the biofilm (Fig.3B).

### Effects of garlic extracts on biofilm formation and bacterial viability

**Figure 4:**
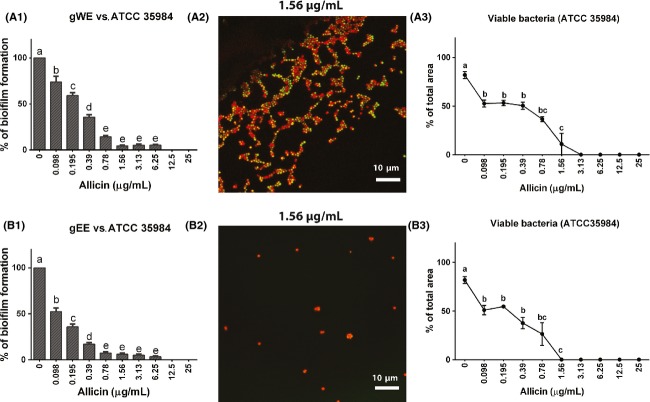
Comparison of the effect of aqueous (gWE) and ethanol (gEE) extracts of fresh garlic standardized for their allicin concentration on *Staphylococcus epidermidis* biofilm formation and bacterial viability in the biofilm. The left panel (A1, B1) show the results of the safranin staining methods as mean percentage (±SEM) of biofilm formation. The middle panel (A2, B2) give examples of this viability staining of the two extracts with the same allicin concentration (1.56 *μ*g/mL allicin). The right panel (A3, B3) show the result (mean ± SEM) obtained with quantitative evaluation of bacterial viability determined by differential viability staining with SYTO® green and propidium iodide and confocal microscopy.

*Staphylococcus epidermidis* ATCC 35984 biofilm was exposed to either an aqueous (water) extract (gWE) or/and ethanol extract (gEE) standardized for their content of allicin. MBIC was 1.56 and 0.78 *μ*g/mL allicin for gWE and gEE, respectively (Fig.4A1 and B1), when using the safranin staining method. Confocal laser scanning microscopy was used to estimate the percentages of nonviable *S. epidermidis* in the controls as well as after treatments with gWE and gEE. It was observed that incubations with gEE containing 1.56 *μ*g/mL allicin resulted in 100% loss of bacterial viability (Fig.4A2 and B2). Differently, bacteria treated with gWE containing 1.56 *μ*g/mL allicin, rendered ∽70% and 60% of nonviable bacteria, and a 100% loss of viability was observed at a concentration of 3.13 *μ*g/mL of allicin in the extract (Fig.4A3 and B3).

### Biofilm architecture determined by confocal laser scanning microscopy

**Figure 5:**
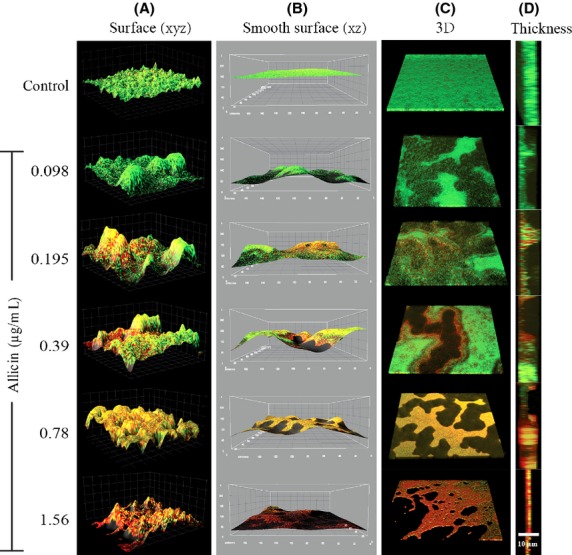
*Staphylococcus epidermidis* (ATCC 35984) biofilm architecture after exposure to increasing concentrations of allicin. CSLM images of *S. epidermidis* biofilm surface and smooth surface from (xz) (A, B); biofilm 3D and thickness images (C, D) after exposure to different allicin concentrations (0.098–1.56 *μ*g/mL). The biofilm thickness decreased from 10.7 (controls) concentration-dependently to 8.6, 7.7, 8.0, 7.9, and 4.7 *μ*m (1.56 *μ*g/mL allicin) with increasing allicin concentrations. Cells were distinguished by staining total bacterial cells with SYTO® green (green) and nonviable bacterial cells with propidium iodide (red).

To obtain further insight into the biofilm structure and density, CLSM was used to obtain surface plot images showing *S. epidermidis* (ATCC 35984) encapsulated in biofilm matrix when bacteria were treated with different concentrations of pure allicin for 24 h (Fig.5). Control samples presented a compact and dense biofilm with a thickness of 10.7 *μ*m. Exposure to allicin resulted in biofilm remodeling, and biofilm thickness decreased rapidly from 10.7 *μ*m (controls) to 8.6 *μ*m after exposure to 0.098 *μ*g/mL allicin, and gradually with increasing allicin concentrations to 4.7 *μ*m after exposure to the highest allicin concentration (1.56 *μ*g/mL). The reduction in biofilm mass was accompanied by a loss a viability of biofilm bacteria. These results support the findings of the MBIC determination.

### Concentration and time-dependent effects of allicin on biofilm-related gene expression

**Figure 6:**
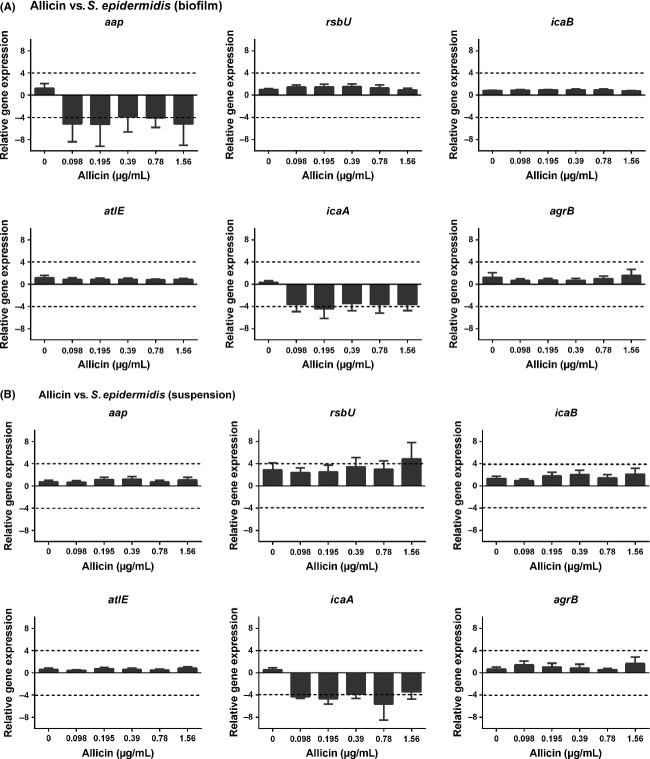
Relative mRNA expression (mean ± SEM) of selected biofilm-related genes in biofilm bacteria (A) and persister cells in suspension (B). Down- and upregulation of gene expression (marked by dashed lines) were considered significant when the relative expression was decreased or increased ≥4 folds.

To gain further insight into the effects of allicin on *S. epidermidis* ATCC 35984 biofilm, the expression of selected genes that mark different phases of biofilm formation was analyzed in parallel in biofilm-adhered bacteria, as well as in nonadhered, persister cells present in the supernatant above the biofilm. In the biofilm-embedded bacteria (Fig.6A), allicin exposure resulted in a downregulation of *aap* and *icaA,* which are associated with the adhesion and bacterial accumulation in a biofilm. In persister cells (Fig.6B), *aap* expression was not influenced by allicin, but *icaA* was downregulated and a concentration-dependent increase in *rsbU*, a bacterial stress factor, was visible.

## Discussion

Garlic is one of the most widely used herbal products and garlic and its extracts are traditionally added to food not only for their taste but also for their food preserving properties. In this study, we aimed to characterize the antibiofilm properties of allicin, the major ingredient of garlic extracts in detail and to compare these effects with gross aqueous and organic extracts of fresh garlic bulbs. Allicin is not present in fresh intact garlic cloves, but is enzymatically formed from its precursor alliin by alliinase within seconds upon crushing garlic cloves by mechanical means (garlic press) or by chewing (Ankri and Mirelman 1999). The concentrations of alliin and subsequently allicin may vary between individual garlic plants, depending on the culture conditions, but an average concentration of 3.4–4.6 mg/allicin per gram fresh garlic can be assumed (Rybak et al. 2004). As a model strain for these investigations, two types of *S. epidermidis* strains were selected, as these strains are isogenic and well characterized. ATCC 12228 grows in planktonic cultures, and ATCC 35984 (also described as RP62A) rapidly forms biofilms under in vitro culture conditions. *Staphylococcus epidermidis* may be considered as a relevant model strain also as it is a facultative pathogen residing on human skin from which is distributed to other individuals directly or transferred to food (Fey and Olson 2010).

As a first step, the MIC of allicin determined in planktonic *S. epidermidis* (strain ATCC 12228) in the broth dilution assay was determined to be 12.5 *μ*g/mL, and confirmed by live–dead staining of bacteria in planktonic growth. These findings are in line with previous investigations (Pérez-Giraldo et al. 2003) and were used to establish the concentration range used in the biofilm experiments. Subsequently, we measured the effect of allicin on ATCC 35984 biofilm formation. In nontreated cultures, *S. epidermidis* biofilm mass increases up to 24 h, which is in agreement with previous studies by Vuong et al. (2003) and Qin et al. (2007) describing also that ∽24 h are necessary for the stable formation of a *S. epidermidis* biofilm. Therefore, we selected this time point to establish the MBIC. The MBIC value is defined as the concentration of an antibiofilm agent that inhibits biofilm formation by >90%. Applying this criterion, a MBIC of 12.5 *μ*g/mL was established when the standard safranin protocol was applied, a value that seems not to differ from the MIC value. This indicates the relative potency of allicin against biofilms as MBIC values of modern antibiotics, such as levofloxacin and vancomycin, show MBIC values of 25 and 50 *μ*g/mL, respectively (Shapiro et al. 2011). To further investigate the effect of garlic on biofilms, we applied a live/dead differential staining and could show that the decrease in biofilm formation was accompanied by a strong bactericidal effect toward biofilm-embedded bacteria, with a 100% loss of viability observed already at a concentration of 3.13 *μ*g/mL allicin. This observation could be confirmed by CLSM analysis of the biofilm architecture, which again showed not only a decrease in biofilm thickness but also an allicin concentration-dependent bactericidal effect on bacteria within the biofilm. This finding is unique, as biofilm-embedded bacteria are generally insensitive to common antibiotics and MIC and MBC values may differ by a factor of 1000 (Taylor 2013).

*Staphylococcus epidermidis* is in the stationary phase after 24 h culture. Lewis (2007) reported that in stationary cultures with biofilm formation, persister cells will appear in the supernatant. Persisters are dormant, nondividing cells, which are seen to be responsible for most biofilm-associated antibiotic tolerance. Shapiro et al. (2011) reported the same phenomenon in *S. epidermidis* RP62A (denoted here ATCC 35984), and showed the reduced antibiotic sensitivity of stationary planktonic bacteria. This is of relevance for biofilm sensitivity on nonviable materials such as food of food processing aids, whereas under in vivo conditions, the phagocytes may be able to opsonize and kill persisters (Lewis 2007). These previous results support our observation that planktonic stationary *S. epidermidis* bacteria were less sensitive to allicin than biofilm bacteria.

CLSM analysis of bacterial biofilm was primarily conducted to assess the effect of allicin on *S. epidermidis* biofilm architecture. Previously Cruz-Villalon and Perez-Giraldo (2011) reported that 4 *μ*g/mL allicin is able to decrease polysaccharide intercellular adhesion (PIA) production in 24 h cultures. CLSM analysis showed a typical tower formation in *S. epidermidis* biofilms already at the lowest concentration of allicin (0.098 *μ*g/mL) and the tower structure remained visible also at the higher concentrations when an increasing percentage of nonviable cells were found. Tower formation is associated with the production of extracellular matrix and the PIA system, requiring viable cells. The fact that this tower formation was observed at all allicin concentrations, support the hypothesis that the decline in biofilm density exerted by allicin is correlated with its concentration-dependent bactericidal effect against biofilm-embedded bacteria, rather than the sole inhibition of biofilm formation via interference with quorum-sensing molecules. PIA production is catalyzed by various glucuronyltransferases regulated by *icaA* in conjunction with *icaD* and other genes, such as *icaB* involved in the processing of PIA (Gerke et al. 1998; Spiliopoulou et al. 2012). These glucuronyltransferases contain cysteine-rich moieties that may be inhibited by allicin (Cruz-Villalon and Perez-Giraldo 2011).

This hypothesis is in agreement with our results of the relative gene expression, where one of the main effects was the downregulation of *icaA*. The concomitantly observed downregulation of *aap* (accumulation-associated protein mediating the intercellular adhesion between bacteria) (Rohde et al. 2005) in the biofilm, that was not observed in the persister cells, suggests the involvement of PIA-independent mechanism in the inhibition of biofilm formation by *S. epidermidis*, but the function and regulation of *aap* are still incompletely understood (Fey and Olson 2010). *AtlE* (autolysin E) mediates the initial attachment of bacteria (Vandecasteele et al. 2003) and generally acts in concert with *agrB* that was not affected by the allicin treatment. The *RsbU* gene is a positive regulator of the activity of sigma factor B, which is the general stress–response factor of Gram+ microorganisms and involved in PIA production as well (Knobloch et al. 2001; Delumeau et al. 2004). *RsbU* was upregulated in persister cells, but not in biofilm-attached cells. *Agr* (accessory gene regulator) is a marker of biofilm dispersion and was not affected by allicin treatment (Vuong et al. 2003; Dai et al. 2012). Taken together, these results demonstrate that allicin affects various stages of biofilm formation in *S. epidermidis*. This is in line with findings in *Pseudomonas aeruginosa* biofilms, indicating that allicin reduced biofilm mass by reducing the adhesion ratio as well as the production of extracellular matrix and the expression of virulence factors (Lihua et al. 2013). In contrast to our finding with allicin in *S. epidermidis*, in *P. aeruginosa* no significant reduction in bacterial growth or loss of bacterial viability was observed and allicin mainly acted as a quorum-sensing inhibitor.

Summarizing the presented results obtained with allicin, it can be concluded that the most prominent effect of allicin is its unprecedented bactericidal effect on *S. epidermidis* embedded in biofilms. The antibacterial activity of allicin has been attributed to an interaction with cellular thiol groups (Fujisawa et al. 2009), a mechanism comparable to the effects of allicin in eukaryotic cells, where allicin interacts with the GSH/GSSG redox system. In gram-positive bacteria, the redox homeostasis is mainly achieved by means of the bacillithiol (BSH) system (Helmann 2011) and further investigations should be devoted to the inhibitory effect on allicin on BSH, which might explain its strong bactericidal effect in biofilms.

Our experimental studies also included an assessment of aqueous (water) (gWE) and organic ethanol (gEE) extract. These extracts were standardized for their allicin content, but contain a variety of other substances present in fresh garlic. A decrease in biofilm formation was observed already at a concentration equal to 0.098 *μ*g/mL allicin, and CLSM analysis showed 100% of nonviable bacteria already at a concentration of 1.56 *μ*g/mL in the gEE group which was lower when compared with gWE and pure allicin (3.13 *μ*g/mL) exposed biofilms. Such a difference was expected and suggests that other components, including ajoene, another sulfuric component and known quorum-sensing inhibitor in garlic, may contribute to the inhibition of biofilm formation (Jakobsen et al. 2012).

In conclusion, our data show a unique bactericidal effect of allicin on *S. epidermidis* biofilms. This effect could only be noted as different assays assessing the antibacterial effects of allicin were combined with differential viability staining followed by confocal microscopy and software aided analysis of the images, which allows the quantification of results. This stepwise assessment can be recommended in general for the assessment of small plant molecules that gain interest as supportive treatment of biofilm-forming bacteria. The presented results underline the opportunities to use garlic or garlic-derived extracts for medical indications such as wound infections, which are often complicated by resistant bacteria like MRSA (Ankri and Mirelman 1999; Cutler and Wilson 2004; Borlinghaus et al. 2014) and in dental medicine where biofilm formation is common (Bachrach et al. 2011). At the same time, garlic and its extracts remain important additives in food preservation increasing the shelf life and the safety of food due to their unique antimicrobial and bactericidal effects.

## Supporting Information

**Figure S1.** Biofilm formation by *S. epidermidis* strains ATCC 35984 (A) and ATCC 12228 (B). **Figure S2.** Time-dependent biofilm formation of *S. epidermidis* (ATCC 35984). **Table S1.** Primers for biofilm-associated genes used in this study.
