# Supplementary material for: Analyzing the antibacterial effects of food ingredients: model experiments with allicin and garlic extracts on biofilm formation and viability of Staphylococcus epidermidis
Source: Food Sci Nutr. 2015 Feb 14;3(2):158–68. doi: 10.1002/fsn3.199 (PMC4376410; doi:10.1002/fsn3.199)
Supplement: Supplementary file 1 [file fsn30003-0158-sd1.docx]

**Supplementary data:**


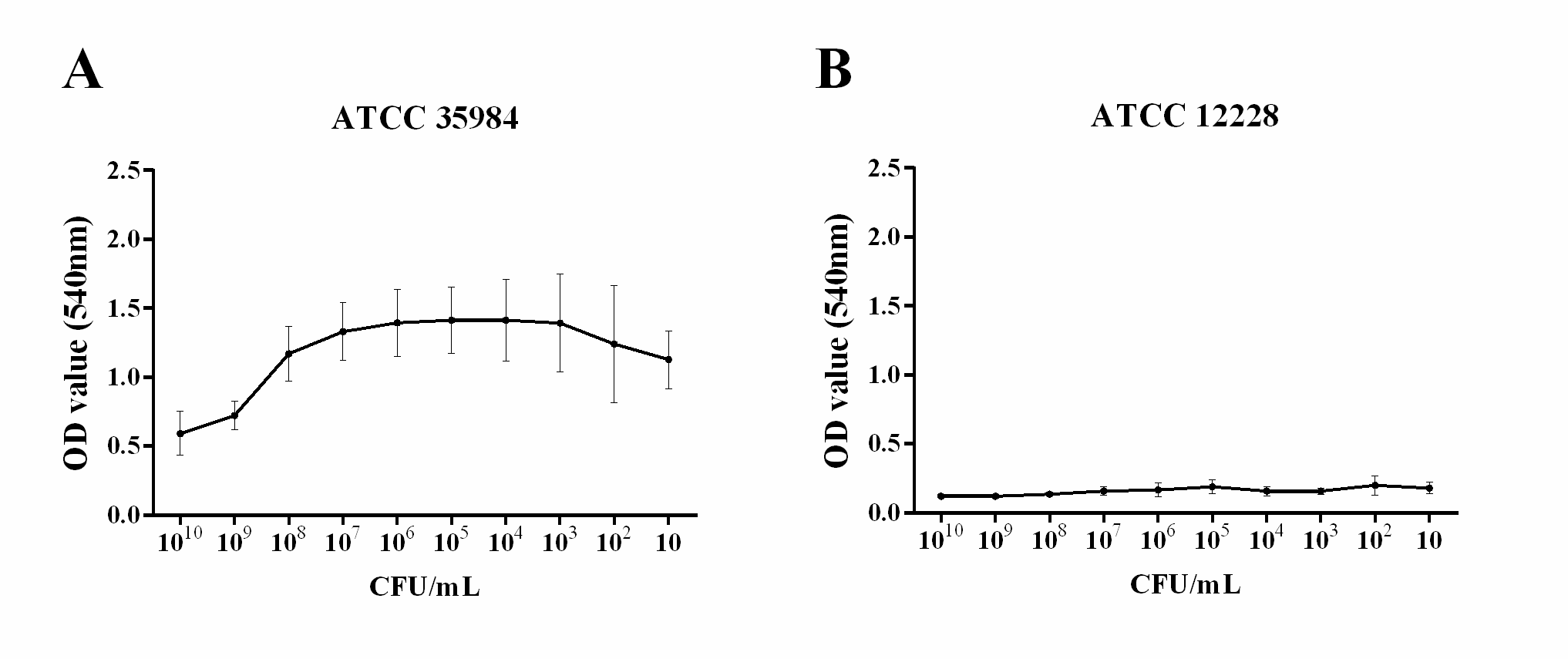


**Supplementary Figure 1.** Biofilm formation by *S. epidermidis* strains ATCC 35984 (A) and ATCC 12228 (B). Data are expressed as mean OD value (± SD) of untreated biofilms from different inoculum sizes measured in three independent experiments using each time six replicates. CFU/mL: Colony-forming unit/mL.


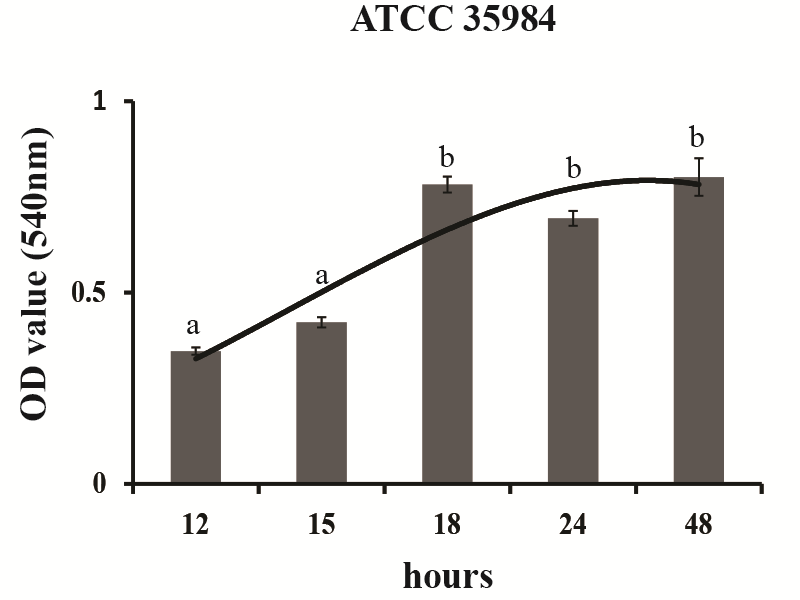


**Supplementary Figure 2.** Time-dependent biofilm formation of *S. epidermidis* (ATCC 35984). Biofilm formation was quantified by safranin staining. Mean OD values (± SEM) were measured at 540 nm wavelength. Different lower-case letters (a, b) indicate significant (*p <* 0.05) differences between time.

| **Gene** | **GenBank** | **Primers sequence (5’-3’)** | **Product size (pb)** | **Reference** |
| --- | --- | --- | --- | --- |
| *hsp60* | AF029245 | F: GTTTTAGCACAATCAATGATTCAG | 491 | [1] |
|  |  | R: GCATCGCCTTCTACTTCATCC |  |  |
| *tpi* | AF269838 | F: CATCTGATAAACCTTCGACAGCTTT | 128 | [2] |
|  |  | R: GTAGCCGTCCAAGTTTTACCAG |  |  |
| *agrB* | AF012132 | F: TTCGTTTAGGGATGCAGGTA | 141 | [3] |
|  |  | R: ATGGCACACGTACAGAGGAT |  |  |
| *atlE* | U71377 | F: TGTCCTGCTTTCACGTATGA | 139 | [3] |
|  |  | R: AGAAACCTTAACCACGTAAA |  |  |
| *icaA* | U43366 | F: AACAAGTTGAAGGCATCTCC | 166 | [4] |
|  |  | R: GATGCTTGTTTGATTCCCT |  |  |
| *icaB* | U43366 | F: AATGGCTTAAAGCACACGAC | 144 | [3] |
|  |  | R:TTTGTCCTTTCCGTAACAGT |  |  |
| *rsbU* | NC002976 | F: TCTCTTCATACAGTCCAT | 172 | [5] |
|  |  | R: ATAGGTTCAGGTATTCCA |  |  |

**Supplementary table 1**: Primers for biofilm-associated genes used in this study.

F: Forward; R: Reverse

**References**

1. Wang X. M., Noble L., Kreiswirth B. N., Eisner W. *et al*., Evaluation of a multilocus sequence typing system for *Staphylococcus epidermidis*. *J. Med. Microbiol.* 2003, *52*, 989–998.
2. Vandecasteele S. J., Peetermans W. E., Merckx R., Van Eldere J., Quantification of expression of S*taphylococcus epidermidis* housekeeping genes with Taqman quantitative PCR during in vitro growth and under different conditions. *J. Bacteriol*. 2001, *183*, 7094–7101.
3. Patel J. D., Colton E., Ebert M., Anderson J. M., Gene expression during *S. epidermidis* biofilm formation on biomaterials. J. Biomed. Mater. *Res. A.* 2012, *100*, 2863–2869.
4. Tormo M. A., Martí M., Valle J., Manna A. C. *et al*., SarA is an essential positive regulator of *Staphylococcus epidermidis* biofilm development. *J. Bacteriol.* 2005, *187*, 2348–2356.
5. Knobloch J. K., Bartscht K., Sabottke A., Rohde H. *et al*., Biofilm formation by *Staphylococcus epidermidis* depends on functional *rsbU*, an activator of the sigB operon: differential activation mechanisms due to ethanol and salt stress. *J. Bacteriol.* 2001, *183*, 2624–2633.
